# A new species of *Periclistus* Foerster, 1869 from China and review of the tribe Diastrophini (Hymenoptera, Cynipoidea, Cynipidae)

**DOI:** 10.3897/zookeys.964.47441

**Published:** 2020-08-27

**Authors:** Yin Pang, Zhiwei Liu*, Cheng-Yuan Su, Dao-Hong Zhu

**Affiliations:** 1 Institute of Evolutionary Ecology and Conservation Biology, Central South University of Forestry & Technology, Shaoshan Road South 498#, Changsha, Hunan 410004, China Central South University of Forestry & Technology Changsha China; 2 Dept. of Biological Sciences, Eastern Illinois University, 600 Lincoln Ave, Charleston, IL 61920, USA Eastern Illinois University Charleston United States of America

**Keywords:** cynipid gall wasp, inquiline gall wasp, molecular phylogeny, Oriental, rose gall

## Abstract

A new species of cynipid gall wasps, *Periclistus orientalis* Pang, Liu & Zhu, **sp. nov.**, is herein described from Hunan, China in the tribe Diastrophini (Hymenoptera: Cynipoidea: Cynipidae). The phylogenetic relationship between *Periclistus* and all the other Diastrophini genera, except the recently described *Xestophanopsis*Pujade-Villar et al., 2019, was analyzed using a fragment of the mitochondrial COI gene and a fragment of the nuclear 28S gene. A taxonomic key to the known genera of Diastrophini and an updated taxonomic key to the known Eastern Palearctic species of *Periclistus* were provided. In addition, an updated checklist of the known species of the genus from the world is given.

## Introduction

Inquiline gall wasps of Cynipidae (Hymenoptera: Cynipoidea) are guests living in the galls induced mostly by other cynipid wasps and occasionally by gall makers of other taxonomic groups, including Cecidomyiidae (Diptera) (Wachi et al. 2011; Ide et al. 2018) and Cecidosidae (Lepidoptera) (Van Noort et al. 2007). According to phylogenetic studies using morphological data, they were considered to have evolved from a single gall-making ancestor that have lost the ability to make galls and were thus all grouped in one tribe, i.e., Synergini (Ritchie 1984; Ronquist 1994; Liljeblad and Ronquist 1998; Ronquist and Liljeblad 2001). However, a comprehensive study on the phylogenetic relationship within Cynipidae based on both morphology and molecular data concluded that inquiline cynipids may have multiple origins, resulting in a significantly revised classification of Cynipidae, particularly with regard to the inquiline members of the family (Ronquist et al. 2015).

Diastrophini is one of the newly established tribes in the updated classification by Ronquist et al. (2015) and currently comprises five genera of gall makers and inquilines exclusively associated with host plants of the Rosaceae family (Pujade-Villar et al. 2016) in the subfamily Rosoideae (Potter et al. 2007). The only species of the tribe that is not associated with a Rosaceae host is *Diastrophus smilacis* reared from *Smilax* sp. (Smilaceae) (Schick et al. 2003). Among the five genera included in Diastrophini, the genera *Diastrophus* Hartig, 1840 and *Xestophanes* Foerster, 1869 consist of gall makers only. *Diastrophus* is widely distributed in the Holarctic and has one known species from Mesoamerica (Nieves-Aldrey et al. 2013) and its members induce galls on species of *Rubus*, *Fragaria*, and *Potentilla* (Palaearctic and Nearctic) (Schick et al. 2003; Melika 2006; Abe et al. 2007) while *Xestophanes* is endemic to Europe in the western Palearctic and the two known species of the genus induce galls on *Potentilla* spp. (Nieves-Aldrey 1994, 2001; Melika 2006). Two other genera of the tribe, *Periclistus* Foerster, 1869 and *Synophromorpha* Ashmead, 1903, are inquilines, using, respectively, galls made by gall makers belonging to different cynipid tribes – species of *Synophromorpha* are associated with galls of *Diastrophus* (Schick et al. 2003) while species of *Periclistus* are associated with galls of *Diplolepis* and *Liebelia* (Cynipidae: Diplolepidini) formed on *Rosa* spp. (Ronquist and Liljeblad 2001; Ronquist et al. 2015). Both *Periclistus* and *Synophromorpha* have a Holarctic distribution (Ritchie 1984; Ritchie and Shorthouse 1987; Ronquist 1994; Ronquist et al. 2015; Pujade-Villar et al. 2015). Although *P smilacis* Ashmead 1896 was reported to be reared from galls of *Diastrophus smilacis* Ashmead, 1896 in Florida, USA, which would also suggest that the species is associated with *Smilax* (Smilaceae), the record seemed to be incorrect (Burks 1979; Pujade-Villar et al. 2016, 2019). The fifth genus, *Xestophanopsis* Pujade-Villar et Wang, 2019, recently described from China is apparently a gall maker associated with Rosaceae host (Pujade-Villar et al. 2019), which needs to be confirmed with rearing data.

The genus *Periclistus* consists of 18 valid species found in the Holarctic region (Pujade et al. 2016; HOL 2018), including five species from the Eastern Palearctic (Pujade-Villar et al. 2016). In the present paper, we describe a new species of the genus from Hunan, China, which is also the first record of the genus from the Oriental region. In the recent study on the phylogeny, evolution and classification of cynipid gall wasps by Ronquist et al. (2015), the tribe Diastrophini was relatively well represented, including at least one species from each genus. The two included *Periclistus* species, i.e., *P. brandtii* and *P pirata* are from the western Palearctic and the Nearctic, respectively. The new species was thus sequenced as a representative from the Eastern Palearctic + Oriental for one mitochondrial gene (COI) and one nuclear gene (28s) and included in an updated phylogenetic analysis of the tribe to examine how the three species are related to each other and the underlying biogeographical implications.

In addition, we also updated the taxonomic key to the species of *Periclistus* Foerster, 1869 from the Eastern Palearctic by Pujade-Villar et al. (2019) and the Oriental region to include the new species and provided a key to the five currently recognized genera of the tribe Diastrophini to facilitate future work on the tribe.

## Materials and methods

Galls collected from April through August were kept in plastic jars with moistened cotton and placed in fine meshed rearing cages. The rearing setup was placed on shelves in ambient environment in the lab and checked daily for emergence. Wasps were collected at emergence and preserved in 100% ethanol, and labeled vials were stored in ultralow freezer at -80 °C for long storage until being retrieved later for preparation for morphological studies or for DNA extraction in molecular studies.

Specimens for conventional morphological examination were air dried at room temperature before being mounted. Specimens mounted to pinned triangle card paper were photographed with Leica M205C microscope system equipped (Leica Inc., Germany) with Leica DMC6200 digital camera attached to a computer.

We follow Ronquist and Nordlander (1989) and Ronquist (1995) for structural terminology, Melika (2006) for measurement definitions, and Harris (1979) for surface sculpture descriptions. Abbreviations:

**F1, F2** the first and second flagellomere, respectively,

**LOL** (lateral-frontal ocelli distance): the distance between anterior and lateral ocelli,

**OOL** (ocellar-ocular distance): the distance from the outer margin of a posterior ocellus to the inner margin of the compound eye, and

**POL** (post-ocellar distance): the distance between the inner margins of the posterior ocelli.

All type specimens are deposited in the Insect Collection, Central South University of Forestry and Technology (**CSUFT**), Changsha, Hunan, China.

Three individuals of the new species were used for DNA extraction. The insects were washed in sterile water before DNA extraction to avoid cross-contamination. Total DNA was extracted from each individual using SDS/proteinase K digestion and phenol-chloroform extraction method as previously described (Zhu et al. 2007). Extracted DNA pellets were air dried, resuspended in 50 µl sterile water, and then stored at 4 °C before being used for PCR and sequencing. For phylogenetic analysis, we chose a specific region of the cytochrome oxidase subunit I gene (COI), which was amplified using the primers HCO-2198 (5’ TAA ACT TCA GGG TGA CCA AAA AAT CA 3’) and LCO-1490 (5’ GGT CAA CAA ATC ATA AAG ATA TTG G 3’) (Folmer et al. 1994), and the ribosome gene 28S, which was amplified using the primers D2F (5’ CGT GTT GCT TGA TAG TGC AGC 3’) and D2R (5’ TCA AGA CGG GTC CTG AAA GT 3’) (Dowton and Austin 2001), or 28Sbout (5’ CCC ACA GCG CCA GTT CTG CTT ACC 3’) and 28SF (5’AGT CGT GTT GCT TTG ATA GTG CAG 3’) primers (Rokas et al. 2002). These two gene fragments were chosen because of their suitability for recovering inter- and intrageneric phylogenies within the Hymenoptera in general and Cynipidae in particular (Rokas et al. 2002) as well as the availability of sequences of the two genes for a reasonable number of congeneric species from public gene sequence depositories. The PCR cycling conditions were: 5 min at 95 °C , followed by 35 cycles of 30 s at 95 °C , 1 min at 46 °C and 1 min at 72 °C, and a final elongation step of 5 min at 72 °C for COI and 5 min at 95 °C, followed by 35 cycles of 30 s at 95 °C, 1 min at 56 °C and 1 min at 72 °C, and a final elongation step of 5 min at 72 °C for 28S. Amplified PCR products were sent to Invitrogen (Shanghai, China) for sequencing. The COI and 28S gene sequences were retrieved from GenBank (https://www.ncbi.nlm.nih.gov/genbank/) for three species of *Diastrophus*, as well as one species for *Synophromorpha*, *Xestophanopsis*, and *Periclistus* respectively. In addition, sequences of the two genes were also acquired from GenBank or by sequencing for *Dryocosmus liui* as outgroup. The final dataset consists of nine species including outgroup (Table 1). Multiple sequence alignment was performed using ClustalW (Thompson et al. 1994) implemented in Mega 7.0 (Kumar et al. 2016) using default parameters. Aligned sequences were then visually edited in Mega 7.0 and trimmed, resulting in a final aligned length of 1133 bp nucleotides, consisting of 670 bps for COI and 490 bps or 1106 bps for 28S.

**Table 1.** List of species included in phylogenetic analysis of Diastrophini relationship based on mitochondrial COI and nDNA 28S. Most sequences were retrieved from GenBank, except for those in bold, which were acquired by sequencing in the present study. Abbreviations for generic names: *Dr* – *Dryocosmus*, *Di* – *Diastrophus*, *Sy* – *Synophromorpha*, *Xe* – *Xestophanes*, and *Pe* – *Periclistus*; for geographical distributions: WP = Western Palearctic, EP = Eastern Palearctic, O = Oriental, and N = Nearctic.

Species	Distribution	COI #		28S #	Reference
*Di. rubi*	WP	DQ012640	DQ012598		Liljeblad (2002)
*Di. potentillae*	N	AY368914	AY368940		Liljeblad (2002)
*Di. turgidus*	N	AY368913	AY368939		Liljeblad (2002)
*Sy. sylvestris*	N	AY368911	AY368937		Liljeblad (2002)
*Xe. potentillae*	WP	AY368912	AY368938		Liljeblad (2002)
*Pe. brandtii*	WP	AF395181	AF395152		Rokas et al. (2002)
*Pe. pirata*	N	DQ012649	DQ012606		Liljeblad (2002)
*Pe. orientalis*	O	**MN633410**	**MN633411**		Present study
*Dr. liui*	EP	MG754067	**MN633412**		Pang et al. (2018), present study

The final dataset was subjected to Mega 7.0 for evaluation of best-fit nucleotide substitution model (Nei and Kumar 2000) using Maximum Likelihood (ML) method with default settings except that we used “very strong” branch swap filter. Phylogenetic analysis was conducted using MrBayes 3.2.6 x64 for Windows (Ronquist et al. 2012) (Bayesian Inference method, BI), assuming a generalized time-reversible (GTR) model with gamma distributed rate variation across sites (+G) based on best fit nucleotide substitution model evaluation described above. For Bayesian analysis, two independent runs were performed with the default priors and MCMC parameters except the following: nst = 6, rates = gamma, MCMC runs comprised 10 million generations sampled at every 1,000 generations with 30% burn-in time. Convergence was achieved as being diagnosed by the average standard deviation of split frequencies between the two independent runs (<0.01) and PSRF values (1 with < 1% deviation). The final tree from both analyses was rooted with *Dryocosmus liui* based on published phylogeny of Cynipidae (Ronquist et al. 2015).

## Taxonomy

### *Diastrophini* Ronquist et al., 2015

Key to genera

**Table:** 

1	Vertex and mesoscutum (Fig. 7) variously sculptured, imbricate to coriaceous. Mesopleuron usually longitudinally striae (Figs 15, 19) and occasionally shining smooth (Fig. 8)	**2**
–	Vertex and mesoscutum smooth, devoid of sculpture (Figs 12, 13). Mesopleuron usually completely smooth without sculpture, and occasionally with very reduced diagonal fine striae	**3**
2	Vertex and mesoscutum mildly to roughly coriaceous, but always entirely punctate setigenous (Figs 5–8, 14, 15). Inquilines of *Diplolepis* and *Liebelia* galls formed on *Rosa* spp. Holarctic	*** Periclistus ***
–	Vertex and mesoscutum mostly mildly coriaceous and scarcely punctate setigenous (Figs 16, 19). Inquilines of *Diastrophus* galls on *Rubus* spp. Holarctic	*** Synophromorpha ***
3	Abdominal terga 3–8 free in both sexes (Fig. 17). Galls mostly on *Rubus* spp., but also on *Fragaria* and *Potentilla*. Mostly Holarctic, with one species from Mesoamerica in Nearctic	*** Diastrophus ***
–	Abdominal terga 3+4 fused in females (Fig. 18); free in males	**4**
4	Antenna of female with 11 flagellomeres, F1 equal or longer than F2; radial cell at most 3.5 times as long as wide, have a weak tarsal tooth. Galls on *Potentilla* spp. Western Palearctic	*** Xestophanes ***
–	Antenna of female with 10 flagellomeres, F1 shorter than F2; radial cell at least 4.0 times as long as broad; tarsal claw with a strong basal lobe. Host plant unknown. Eastern Palearctic	*** Xestophanopsis ***

### *Periclistus* Forster, 1869

#### Periclistus orientalis

Taxon classificationAnimaliaHymenopteraCynipidae

Pang, Liu & Zhu sp. nov.

3EA84E9E-3CB7-5419-A10C-ED8CFC50C0B6

http://zoobank.org/77D32C97-1A16-4FE6-9A17-EC87B42749EB

Figures 1–6 , 7–11

##### Type materials.

***Holotype***: ♀ (CSUFT), China, Hunan Province, Zhuzhou City, 27.83N, 113.13E, reared in 2011-V-10-20 from galls collected in 2011-IV, leg. Xiao-Hui Yang; ***Paratypes***: 4♀♀, 2♂♂ (CSUFT), collection data and locality same as holotype.

##### Etymology.

The species epithet is derived from Latin *orient*, meaning east, to suggest the type locality from the Oriental region.

##### Diagnosis.

*Periclistus orientalis* can be distinguished from the other congeneric species in the Eastern Palearctic using the taxonomic key provided herein. Below we provide more detailed comparison of the new species with the two very similar species, i.e., *P setosus* and *P capillatus*.

**Figures 1–6.**
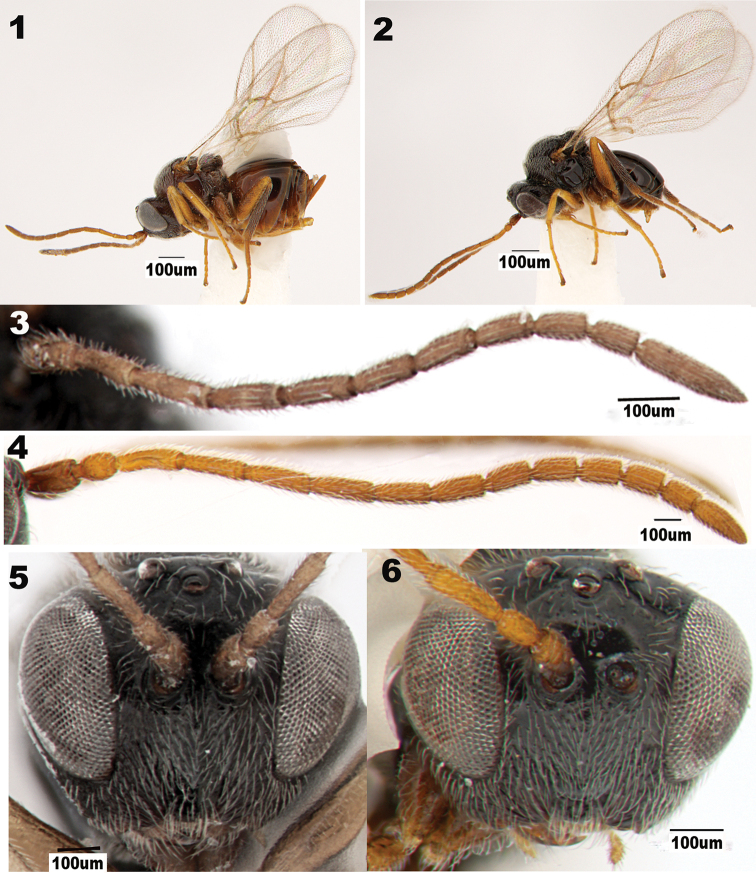
*Periclistus orientalis* sp. nov. **1** general habitus (♀) **2** general habitus (♂) **3** antenna (♀) **4** antenna (♂) **5** head in anterior view (♀) **6** head in anterior view (♂).

**Figures 7–11.**
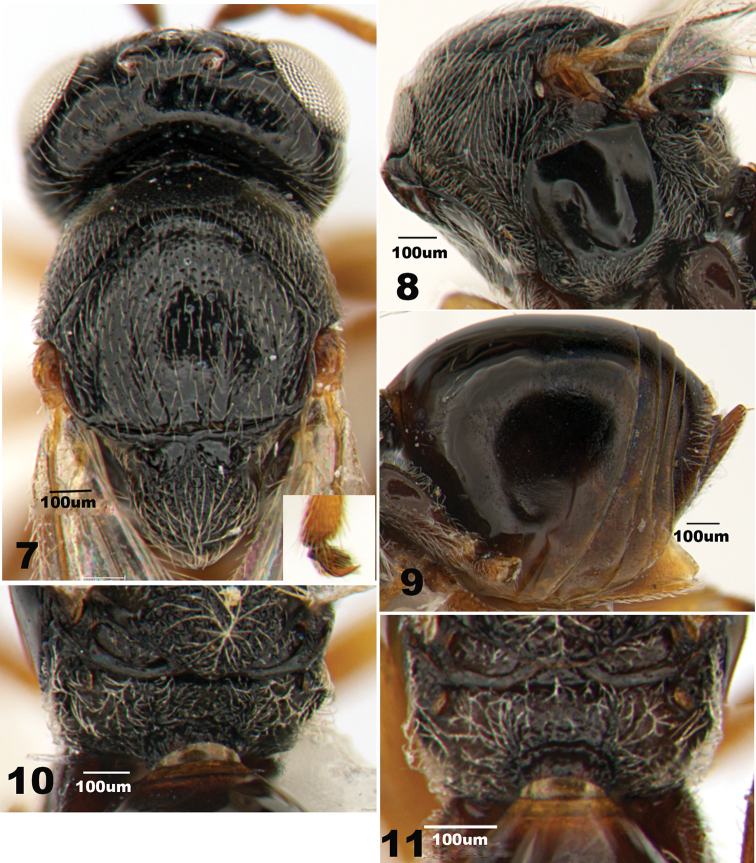
*Periclistus orientalis***7** head and mesosoma in dorsal view (♀, similar in ♂) **8** mesosoma in lateral view (♀) **9** metasoma in lateral view (♀) **10** propodeum in dorsal view (♀) **11** propodeum in dorsal view (♂).

*Periclistus orientalis* sp. nov. is similar to *P setosus*, but differs from the latter in the lower face with striae radiating from clypeus reaching eyes and antennal socket in the new species, whereas in *P setosus* striae of lower face not reaching eyes and antennal socket (Fig. 5); notauli distinctly present in posterior one third of scutum and medial sulcus absent in the new species, whereas complete and distinctly in *P setosus* (Wang et al., 2012) (Fig. 7); lateral surface of pronotum entirely coriaceous with evenly distributed dense setigerous punctures (Fig. 8) in the new species, but in the latter lateral surface of pronotum glabrous, with sparse setigerous punctures ventrolaterally. The new species is also similar to *P capillatus* Belizin, 1968. It differs from *P capillatus* in the mesoscutum glabrous with piliferous punctures and dense appressed pubescence in the new species, whereas with piliferous points and sparse pubescence in *P capillatus* (Fig. 7); notauli distinctly present in posterior one third of scutum and medial sulcus absent in the new species, whereas incomplete or very weakly impressed anteriorly in *P capillatus* (Fig. 7); fused metasomal tergites T2+T3 anterolaterally with a patch of sparse white setae, mostly smooth except for minute punctures on laterally posterior half and a narrow band of punctures along posterior margin, whereas in the latter metasomal tergites fused (T2+T3) smooth, with an anterolateral patch of white setae, and the subsequent segments glabrous with micropunctures (Figs 1, 9).

##### Description.

**Female: *Body*** length 2.7–2.8mm (*N* = 5).

***Coloration*.** Head completely black. Antenna uniformly light brown. Front and middle legs reddish brown, except coxa and claw black; hind legs black, except tarsomeres 1 and 5 reddish brown. Mandible and maxilla reddish brown, labial palpi light brown. Mesosoma black; metasoma mostly reddish brown in anterior half, and dark in dorsal half. Ventral spine of hypopygium reddish brown.

***Antenna*** filiform with ten flagellomeres, slightly tapering toward apex; pedicel 1.67 times as long as broad; relative lengths of scape, pedicel and F1-F10: 9:5:10:10:10:9:8:8:7:6:6:13 (Fig. 3).

***Head*** coriaceous, with sparse setae, 2.0 times as broad as long in dorsal view, 1.24 times as wide as high and slightly broader than mesosoma in dorsal view. Gena delicately coriaceous and not broadened behind eyes. Malar space 0.27 times as high as height of eye. Lower face with striae radiating from clypeus and reaching eyes and antennal socket, entirely densely punctate with white, long, and appressed setae; median area slightly elevated, delicately coriaceous, lateral carinae bordering median area complete from clypeus to antennal socket and about as strong as radiating striae on lateral areas of lower face. Clypeus inversely trapezoid, ventral margin straight, and delicately coriaceous with dense long setae; anterior tentorial pits indistinct; epistomal sulcus and clypeo-pleurostomal lines indistinct. Transfacial distance longer than height of eye; distance between inner margin of eye and outer rim of antennal torulus slightly longer than distance between antennal toruli, all larger than diameter of torulus (Fig. 5). Ratios of POL/OOL, POL/LOL, and LOL/OOL1.3, 1.8 and 0.7, respectively. Frons, vertex and gena behind eyes, and postgena with sparse setigerous punctures; setae long and white. Frons coriarious and smooth, with scattered punctures. Vertex smooth and evenly punctate with long setae (Fig. 7).

***Mesosoma*** longer than high in lateral view and with white setae. Pronotum median length nearly one third of length of outer lateral margin; anterior lateral depressions medially separated broadly from each other, laterally open, continuing to a distinct furrow; posterior rim of anterior lateral depressions extending dorsally to reach posterior margin of pronotum, distinctly separating anterior plate from lateral pronotal areas. Anterior plate of pronotum delicately coriaceous, posteriorly with shallowly punctate and sparsely setose (Fig. 7); lateral pronotal areas coriaceous, entirely densely punctate with appressed long setae, without glabrous ventral nude area (Fig. 8). Mesoscutum smooth and shiny, slightly broader than long, distinctly depressed anteriorly, with evenly dispersed piliferous punctures; anteroadmedian signum absent, notauli distinctly present in posterior one third of scutum, medial sulcus absent, parapsidal signa present in posterior half and absent anteriorly (Fig. 7). Scutellar foveae large, deeply impressed, glabrous, separated by a broad median carina (Fig. 7). Mesoscutellum about as broad as long, rugose and foveolate with sparse, appressed setae (Fig. 7). Mesopleuron distinctly higher than broad, glabrous and shining, devoid of striation and pubescence, except for pubescence along ventral margin (Fig. 8); mesopleural triangle glabrous, not separated from rest of mesopleuron by a ventral carina. Metapleural sulcus reaching mesopleuron in upper one fourth of its height; metapleuron rugulose with long setae; metanotum slightly overhanging. Lateral propodeal carinae distinct and evenly curved outwards; median propodeal area rugose foveolate; lateral propodeal areas with dense setae (Fig. 10).

***Forewing*** with distinct veins R+Sc, R1+Sc, R1, M, M+Cu1, Cu1, Cu1b, Cu1a, 2r and Rs+M; areolet distinct and large; radial cell closed, 3.3 times as long as wide; all visible veins dark brown (Fig. 1).

***Metasoma*** nearly as long as head and mesosoma combined, distinctly longer than height in lateral view, distinctly punctate posteriorly. Metasomal tergites 2+3 with some setae ventrally. Prominent part of ventral spine of hypopygium very short (Fig. 9).

**Male**: Similar to female, but different as follows. Antenna with 12 flagellomeres, pedicel 2.5 times as long as broad. F1 strongly curved medially. Relative lengths of scape, pedicel and F1-F12: 7:5:13:12:7:7:6:6:5:5:5:5:4:7 (Fig. 4). Second and third metasomal tergites not fused, separated by a suture (Fig. 2).

##### Biology.

All specimens were reared from galls collected from *Rosa multiflora*, and the galls were very similar in morphology to those made by *Diplolepis japonica*: fleshy and spherical with pointed spikes on top, pinkish green to greenish yellow in color, and located on rachis or central vein of leaflets of both upper and under sides (Fig. 20). Nonetheless, the identity of the host gall maker remains reclusive since our rearing yielded no specimen of the putative gall maker. The galls were collected from April through August, and specimens of *P. orientalis* emerged in early May from galls collected in April.

##### Distribution.

Known from Zhuzhou City, Hunan Province, China.

The known species of *Periclistus* in the Eastern Palearctic can be identified using the following taxonomic key modified from Pujade-Villar et al. (2016) to accommodate the new species.

### Taxonomic Key to Eastern Palearctic species of *Periclistus* Foerster, 1869

**Table:** 

1	Forewing with a small clouded macula posterior to anterior margin near apex of radial cell; radial cell of forewing long, ca 4.0 times as long as wide, and open distally	**2**
–	Forewing hyaline; radial cell of forewing short, ca 3.0 times as long as wide, and partially closed or closed with inconspicuous submarginal vein	**3**
2	Notauli present anteriorly, weakly impressed; and metasoma reddish brown (Distribution: Japan: Honshu, Shikoku and Kyushu)	***P natalis***
–	Notauli absent; and metasoma dark brown (Distribution: Japan: (Honshu, Shikoku and Kyushu)	***P quinlani***
3	Notauli completely absent. (Distribution: China: Qinghai)	***P qinghainensis***
–	Notauli present, complete or incomplete	**4**
4	Fronts and vertex without fine piliferous punctures; F1 slightly shorter than F2; notauli incomplete, absent to very weakly impressed in anterior 2/3 to 3/4 of scutum. (Distribution: Russia: Primorie (in the Far East) and China: Henan, Shaanxi)	***P. capìllatus***
–	Fronts and vertex with fine piliferous punctures; F1 is equal to F2 in length; notauli complete	**5**
5	Lower face with striae radiating from clypeus not reaching eyes and antennal socket; notauli complete and deeply impressed throughout, narrow anteriorly and relatively broadened posteriorly; lateral surface of pronotum glabrous, with sparse setigerous punctures ventrolaterally. (Distribution: China: Zhejiang, Fujian)	***P setosus***
–	Lower face with striae radiating from clypeus reaching eyes and antennal socket; notauli distinctly present in posterior one third of scutum and medial sulcus absent; lateral surface of pronotum entirely coriaceous with evenly distributed dense setigerous punctures (Fig. 8). (Distribution: China: Hunan)	***P. orientalis***

## Discussion

The Diastrophini tribe consists of gall inducers and inquilines of galls, which are all associated with Rosaceae plants belonging to the supertribe Rosodae (Potter et al. 2007), except for the newly described monotypic genus *Xestophanopsis*, whose biology is not yet known (Pujade-Villar et al. 2019). The major morphological difference between the gall-maker and inquiline genera of the tribe is the lack of any kind of sculpture on upper face, vertex, and mesoscutum in the gall maker genera, a feature also shared by *Xestophanopsis*. The genus *Diastrophus* is unique compared to the other genera of the tribe in having metasomal terga 2 and 3 free in female (Fig. 17), not fused as in the other genera of the tribe while *Xestophanes* differs from all other genera of the tribe by having a rather reduced basal lobe on tarsal claw, rather than a well-developed one (Melika 2006, Ritchie 1984, Pujade-Villar et al. 2019). *Xestophanes* is further separated from *Xestophanopsis* by having eleven flagellomeres in female antenna, rather than having ten as in the latter (Pujade-Villar et al. 2019). On the other hand, the two inquilinous genera, *Synophromorpha* and *Periclistus*, are morphologically very similar. Ritchie and Shorthouse (1987), in their revision of *Synophromorpha*, listed several diagnostic features separating *Periclistus* from the former, including mesoscutum coriaceous; notauli weaker, not percurrent, and not broadened posteriorly or with anterior pits; ventral margin of subalar triangle with row of setigerous punctures; radial cell closed; male A3 usually strongly notched and broadened distally. However, these differences are either hard to define and become less obvious when the eastern Asian *Periclistus* (Fig. 7) species are included in the comparison (Abe 1998). Abe (1998) also mentioned that the two genera differ in how the mesoscutellum extended posteriorly, but we have observed no difference regarding this feature by comparing *P. brandtii* (Fig. 15) and *S. sylvestris* (Fig. 19). Biologically, the two genera have different host plant and host gall associations – all *Synophromorpha* species with known host data are guests in the galls made by *Diastrophus* species on *Rubus* plants (Ritchie and Shorthouse 1987; Abe 1998; Wachi et al. 2013) while all *Periclistus* species with available host data are guests of galls made by *Diplolepis* spp. and *Liebelia* spp. (Diplolepidini, Cynipidae) on *Rosa* plants (Ritchie 1984; Ronquist and Liljeblad 2001; Ronquist et al. 2015; Pujade-Villar et al. 2016). Therefore, it was considered more suitable to retain these two genera as separate despite their close morphological affinity (Abe 1998). Our phylogenetic analysis and genetic distance comparison, although based on limited molecular data available, provide support for this proposition. Phylogenetically the two genera do not form a monophyletic clade (Fig. 21; Ronquist et al. 2015). The pairwise COI sequence distance between *Sy. sylvestris* and *P pirata*, and *P. orientalis* are 19% and 20%, respectively, which are considerably higher than those between *Sy. sylvestris* and species of the gall making genera of the Diastrophini tribe (Table 2). Furthermore, the two genera seem to be reliably separated morphologically as well, by the general lack of setae and weaker sculpture on head, lateral sides of pronotum, mesoscutum and mesopleuron in *Synophromorpha* (Figs 16, 19) as compared to *Periclistus* (Figs 5–8, 14, 15).

**Table 2.** Pair-wise COI sequence distance between four Diastrophinii genera, Periclistus (Pe.), Diastrophus (Di.), Xestophanes (Xe.), and Synophromorpha (Sy.). *Xestophanopsis* is not included in the comparisons because of lack of data and specimens.

	***Di. potentillae***	***Di. turgidus***	***Di. rubi***	***Pe. orientalis***	***Pe. pirata***	***Pe. brandtii***	***Sy. sylvestris***	***Xe. potentillae***
*Di. potentillae*								
*Di. turgidus*	0.13							
*Di. rubi*	0.12	0.10						
*Pe. orientalis*	0.23	0.25	0.22					
*Pe. pirata*	0.21	0.21	0.19	**0.17**				
*Pe. brandtii*	0.19	0.21	0.17	**0.15**	**0.12**			
*Sy. sylvestris*	0.13	0.14	0.11	0.20	0.19	0.14		
*Xe. potentillae*	0.14	0.09	0.10	0.19	0.17	0.15	0.07	
*Dr. liui*	0.22	0.22	0.24	0.28	0.24	0.22	0.19	0.18

Key: numbers in bold indicate pairs of congeners of *Periclistus*; numbers in grey block indicate pairs between a *Periclistus* species and a species of another genus.

**Figures 12–19.**
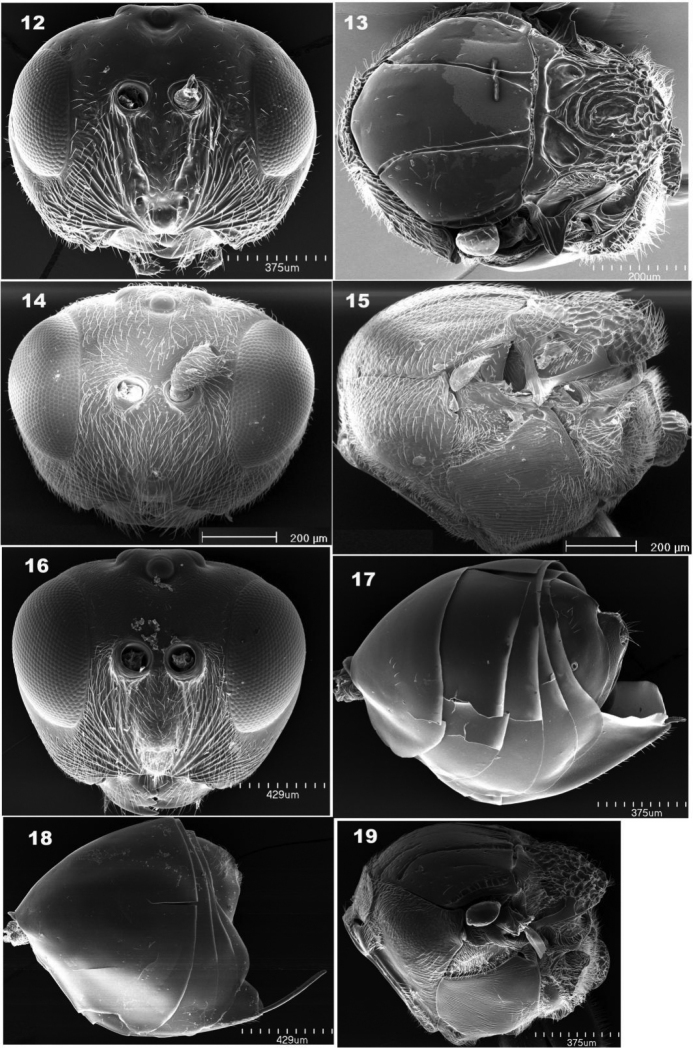
SEM images of representative Diastrophini species **12***Diastrophus nebulosus* head in anterior view (♀) **13***Diastrophus nebulosus* mesosoma in dorsal view (♀) **14***Periclistus brandtii* head in anterior view (♀) **15***Periclistus brandtii* mesosoma in lateral view (♀) **16***Synophromorpha sylvestris* head in anterior view (♀) **17***Diastrophus nebulosus* metasoma in lateral view (♀) **18***Xestophanes potentillae* metasoma in lateral view (♀) **19***Synophromorpha sylvestris* mesosoma in lateral view (♀).

There exists confusion about the number of valid known species in *Periclistus*, ranging from 14 (Penzes et al. 2012; Pujade-Villar et al. 2016) to 18 (HOL, the Hymenoptera Online database, 2018). Apparently, the latter was uninformed of the fact that several species have been transferred to other genera since the initial descriptions. Below we provide an update of species list for the genus, including the species described and recombination published since Penzes et al. (2012). Information and sources on detailed distribution, host gall making species, and host plants are found in Ritchie (1984) and summarized in Penzes et al. (2012). *Periclistus idoneus* Belizin, 1973 was subsequently transferred to *Aulacidea* by Pujade-Villar et al. (2016) and thus is not included herein (* denotes synonymy and recombination suggested by Ritchie (1984), NA – Nearctic, PA – Palearctic, and O – Oriental).

1. *P arefactus* McCracken & Egbert, 1922 NA

2. *P. brandtii* (Ratzeburg, 1831) PA

3. *P californicus* Ashmead, 1896 NA

4. *P caninae* (Hartig, 1840) PA

5. *P capillatus* Belizin, 1968 PA

6. *P mongolicus* Belizin, 1973 PA

7. *P natalis* Taketani & Yasumatzu, 1973 PA

8. *P obliquus* Provancher, 1888* NA

9. *P. orientalis* sp. nov. O

10. *P piceus* Fullaway, 1911 NA

11. *P pirata* (Osten-Sacken, 1863) NA

12. *P qinghaiensis* Pujade-Villar et al., 2015 EP

13. *P quinlani* Taketani & Yasumatzu, 1973 PA

14. *P semipiceus* (Harris, 1841)* NA

15. *P smilacis* Ashmead, 1896* NA

Ritchie (1984), in his dissertation on inquiline Cynipidae, conducted an extensive revision of the genus *Periclistus*, and proposed synonymy and recombination for several species (indicated in the above list with *) in the genus, including the transfer of *P semipiceus* to *Diplolepis* and *P obliquus* Provancher to *Eumayria*, and considered *P smilacis* a junior synonym of *P pirata*. In addition, six species were also described as new in the dissertation (named by Ritchie and Shorthouse). Unfortunately, the work has not been published and therefore are not considered valid taxonomic changes until future publication.

Within *Periclistus*, the new species is easily grouped together with its congeners from the Eastern Palearctic in that they all have entirely smooth and shiny mesopleuron without striae, mesoscutum smooth and moderately punctate setigerous (Pujade-Villar et al. 2015), suggesting that the Eastern Palearctic species may form a monophyletic lineage, which nonetheless needs to be tested based on formal phylogenetic analysis.

**Figure 20.**
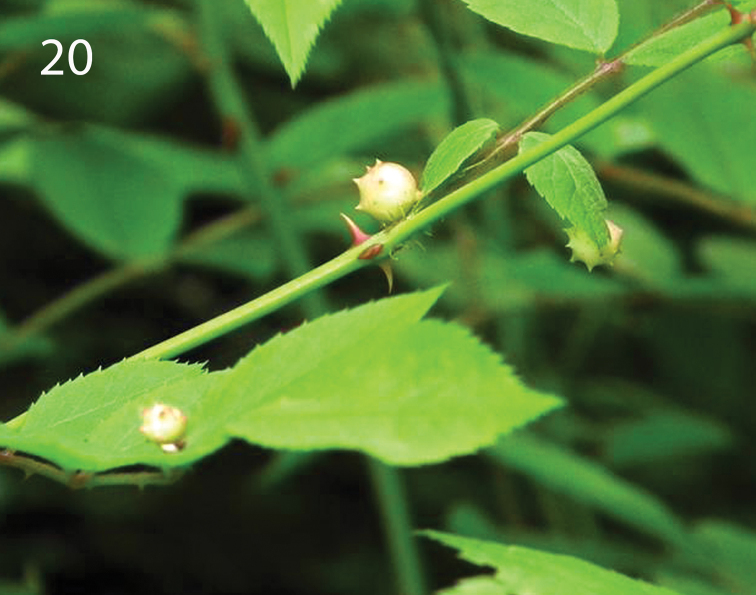
Galls on *Rosa multiflora*, from which specimens of *Periclistus orientalis* were reared.

**Figure 21.**
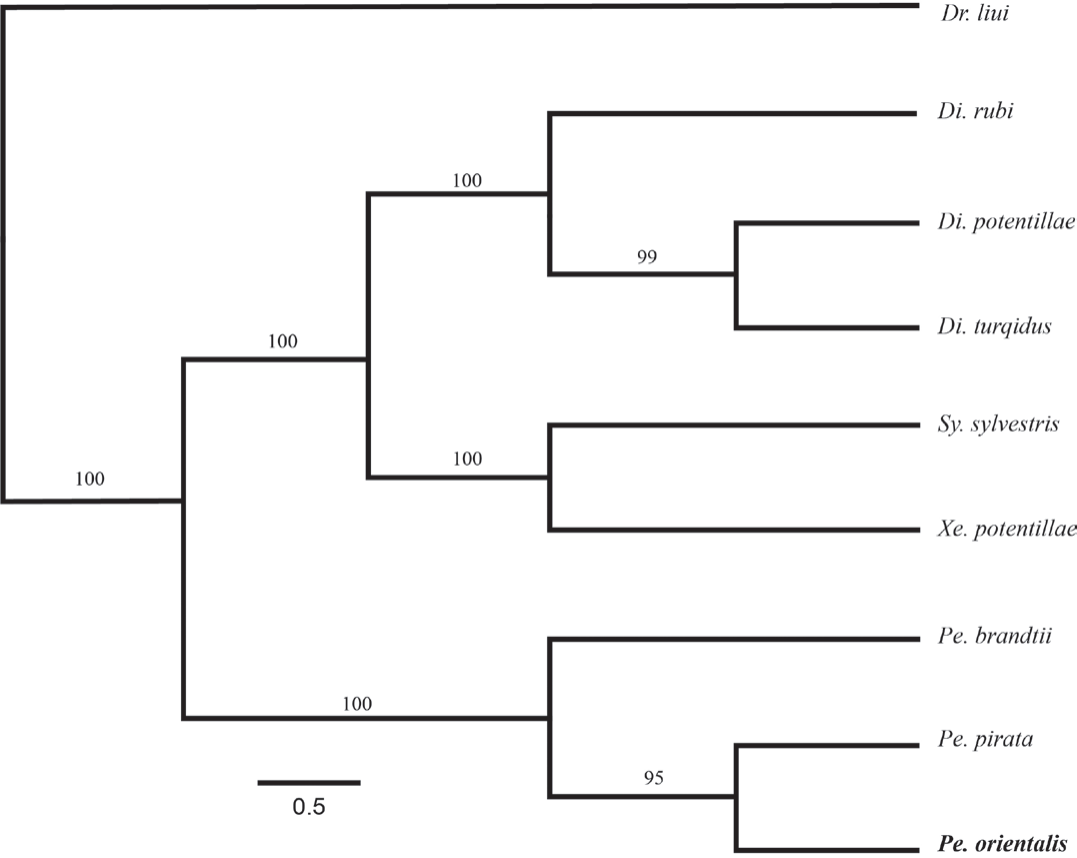
Phylogenetic relationship of Diastrophini species based on COI and 28S sequences resolved using with MrBayes 3.2.6 (Ronquist et al. 2012). Two independent MCMC runs were run with the following parameters: 10 million gens, nst = 6, rates = gamma, sample frequency = 1/1,000, burn-in = 30%, and otherwise default. The length of the branches is drawn to scale of genetic distance and the number over branches is posterior probability. Abbreviations for generic names: *Dr* – *Dryocosmus*, *Di* – *Diastrophus*, *Sy* – *Synophromorpha*, *Xe* – *Xestophanes*, and *Pe* – *Periclistus*.

With the inclusion of *P. orientalis* in our analysis based on COI and 28S sequences, the resulting phylogenetic tree (Fig. 21) is consistent with the multiple gene tree of Ronquist et al. (2015) regarding the Diastrophini. In our result, *P. orientalis* as a representative species from the Eastern Palearctic + Oriental is shown to be more closely related to the eastern Nearctic *P pirata* than with *P. brandtii* from the western Palearctic, which may suggest the Eastern Asia-Eastern North America disjunct distribution frequently observed in flowering plants (Xiang et al. 1998; Wen 1999) and other organismal groups, including insects (Nordlander et al. 1996; Ren et al. 2019). However, the suggestion should be taken with caution since we only sampled one single species from each region, and future phylogenetic analysis with more dense species sampling is needed to test this hypothesis.

## Supplementary Material

XML Treatment for Periclistus orientalis
